# Case Report of Two Independent Moroccan Families with Syndromic Epidermodysplasia Verruciformis and STK4 Deficiency

**DOI:** 10.3390/v16091415

**Published:** 2024-09-05

**Authors:** Assiya El Kettani, Hind Ouair, Farida Marnissi, Jalila El Bakkouri, Rémi Chevalier, Lazaro Lorenzo, Halima Kholaiq, Vivien Béziat, Emmanuelle Jouanguy, Jean-Laurent Casanova, Ahmed Aziz Bousfiha

**Affiliations:** 1 Laboratory of Clinical Immunology-Inflammation and Allergy (LICIA), Faculty of Medicine and Pharmacy, Hassan II University, Casablanca 20250, Morocco; jalilaelbakkouri@gmail.com (J.E.B.); halima.kholaiq-etu@etu.univh2c.ma (H.K.); profbousfiha@gmail.com (A.A.B.); 2 Laboratory of Bacteriology, Virology and Hospital Hygiene, Ibn Rochd University Hospital, Casablanca 20250, Morocco; 3 Laboratory of Bacteriology and Virology, Faculty of Medicine and Pharmacy, Hassan II University, Casablanca 20250, Morocco; ouairhind@gmail.com; 4 Laboratory of Pathological Anatomy, Ibn Rochd University Hospital, Hassan II University, Casablanca 20250, Morocco; faridamarnissi@hotmail.fr; 5 Immunology Laboratory, Ibn Rochd University Hospital, Casablanca 20250, Morocco; 6 Laboratory of Human Genetics of Infectious Diseases, Necker Branch, Institut National de la Santé et de la Recherche Médicale (INSERM), 75015 Paris, France; remi.chevalier@institutimagine.org (R.C.); lazaro.lorenzo-diaz@inserm.fr (L.L.); vivien.beziat@inserm.fr (V.B.); emmanuelle.jouanguy@inserm.fr (E.J.); casanova@mail.rockefeller.edu (J.-L.C.); 7 Imagine Institute, Paris Cité University, 75015 Paris, France; 8 Laboratory of Human Genetics of Infectious Diseases, Rockefeller Branch, Rockefeller University, New York, NY 10065, USA; 9 Howard Hughes Medical Institute, Chevy Chase, MD 20815-6789, USA; 10 Clinical Immunology and Infectious Pediatrics Department, Abderrahim Harouchi Hospital-Ibn Rochd University Hospital, Casablanca 20250, Morocco

**Keywords:** epidermodysplasia verruciformis, human papillomavirus, STK4 deficiency, CD4^+^ T cells

## Abstract

Epidermodysplasia verruciformis (EV) is a rare genodermatosis caused by β-human papillomaviruses (HPV) in immunodeficient patients. EV is characterized by flat warts and pityriasis-like lesions and might be isolated or syndromic, associated with some other infectious manifestations. We report here three patients from two independent families, with syndromic EV for both of them. By whole exome sequencing, we found that the patients carry new homozygous variants in STK4, both leading to a premature stop codon. STK4 deficiency causes a combined immunodeficiency characterized by a broad infectious susceptibility to bacteria, viruses, and fungi. Auto-immune manifestations were also reported. Deep immunophenotyping revealed multiple cytopenia in the three affected patients, in particular deep CD4^+^ T cells deficiency. We report here the fourth and the fifth cases of the syndromic EV due to STK4 deficiency.

## 1. Introduction

Epidermodysplasia verruciformis (EV) is a genodermatosis characterized by pityriasis versicolor and flat wart-like lesions. EV is a rare disease, with around 500 cases reported so far, and is the consequence of β-papillomavirus (β-HPV) infection [1,2]. Patients with EV, after decades, have a high risk of developing actinic keratosis and non-melanoma skin cancer (NMSC), particularly cutaneous squamous cell carcinoma (cSCC), cSCC in situ (Bowen’s disease) and, to a lesser extent, basal cell carcinoma (BCC). NMSC typically occurs in lesions exposed to the sun [3]. EV can be isolated or syndromic, including additional clinical features, mainly infectious [3]. Since the 1940s, it has been hypothesized that EV might also be a genetic disease [4]. In 2002, EVER1 and EVER2 were identified as the first two genes underlying isolated EV [5]. More recently, CIB1 deficiency was reported in patients with isolated EV and as a new partner of EVER1 and EVER2 [6]. EVER1, EVER2, and CIB1 deficiencies are believed to result in a keratinocyte intrinsic deficiency, underlying exquisite susceptibility to β-HPVs [6]. In contrast, syndromic EV has been associated with T cell deficiencies, sharing CD4^+^ T cell lymphopenia [7,8,9,10]. These etiologies of atypical EV include loss-of-function (LOF) mutations of RHOH [11], STK4 [12,13,14], CORO1A [15], FLT3LG [16], TRAC [17], DCLRE1C (encoding the ARTEMIS protein) [18], DOCK8 [19,20], RASGRP1 [21], LCK [22], TPP2 [23], and ITK [24]. Here, we report two novel cases of STK4 deficiency associated with atypical EV. 

## 2. Clinical Reports

### 2.1. Clinical Phenotypes and Genotypes

#### 2.1.1. Family 1

From birth to 10 years old, the proband (P1) clinical history was unremarkable. He received all the childhood vaccines (BCG, MMR, diphteria, tetanus, poliovirus, and HBV) without any complications. However, at the age of 10, he presented recurrent apyretic diarrhea episodes, with no pathogen identified. At the age of 12 years old, the P1 growth curve presented a negative deviation from the norm. He had no cardiac clinical symptoms and no electrocardiogram or echocardiographic abnormalities.

**Figure 1:**
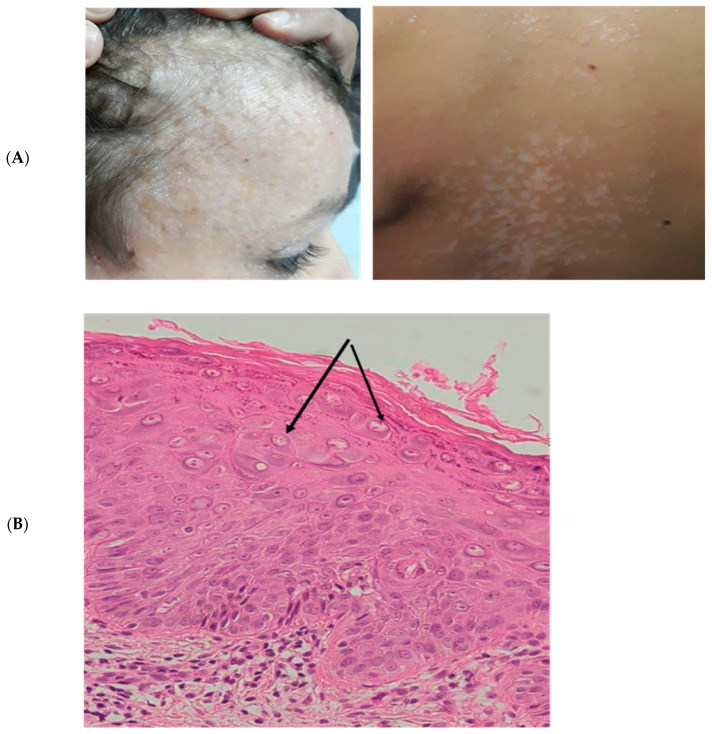
(**A**). Pictures of the lesions on the face and the back of P1. (**B**). Histological study of Punch skin biopsy showed hyperkeratosis and parakeratosis, mild acanthosis, and the presence of koilocytes, keratinocytes with pale-stained cytoplasm in the upper epidermis associated with high levels of intranuclear viral replication. The cytoplasm of the affected cells stains pale blue which is pathognomonic of epidermodysplasia verruciformis lesions without any sign of malignancy (arrows).

P1 was also admitted to pediatrics consultation for generalized flat warts and pityriasis versicolor-like skin lesions that progressed from the age of 6 years old (Figure 1A). The lesions were initially localized on the face and then generalized to the neck, trunk, arms, and the back.

The histology analysis showed hyperkeratosis and parakeratosis, mild acanthosis, and the presence of koilocytes, keratinocytes with pale-stained cytoplasm in the upper epidermis associated with high levels of intranuclear viral replication (Figure 1B). All together, these observations suggested a diagnosis of epidermodysplasia verruciformis. The genotyping of HPV by PCR confirmed the diagnosis, with the identification of HPV5 in a punch biopsy of a lesion from his right arm. 

His 16-year-old sister (P2) did not develop EV or any HPV-related skin disease, but she had presented with recurrent low respiratory tract infections since the age of 3 years old with no pathogen identified. She also received all the childhood vaccines (BCG, MMR, diphteria, tetanus, poliovirus, and HBV) without any complication, and her growth curve presented a deviation from the norm as well.

No secondary immunodeficiency was noted in both siblings and no auto-immune manifestation was reported so far. In terms of treatment, both siblings have been treated with intravenous immunoglobulins substitution as well as antibiotic prophylaxis against opportunistic infections (Trimethoprim/Sulfamethoxazole). In addition, P1 had a treatment with imiquimod application on the skin, and skin protection from UV radiation and the skin lesions improved.

#### 2.1.2. Family 2

**Figure 2:**
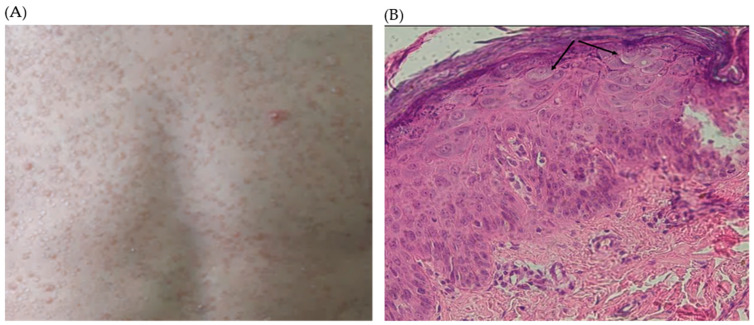
(**A**). Pictures of the lesions on the face and the hand of P3. (**B**). Histological study of Punch skin biopsy showed hyperkeratosis and parakeratosis, mild acanthosis, and the presence of koilocytes, keratinocytes with pale-stained cytoplasm in the upper epidermis associated with high levels of intranuclear viral replication. The cytoplasm of the affected cells stains pale blue, which is pathognomonic of epidermodysplasia verruciformis lesions without any sign of malignancy (arrows).

An 8-year-old girl (P3) from parents in a consanguineous marriage presented in pediatric consultation for profuse pityriasis versicolor-like skin lesions along the upper and lower limbs, the trunk, and the face (Figure 2A). These lesions have been evolving since the age of 3 years old. Skin histology of a lesion was typical of EV lesions (Figure 2B).

By performing PCR on a sample from the lesion on the right forearm of the patient, we identified HPV8, confirming the EV diagnosis. She also had recurrent respiratory infections and diarrhea, with stature weight repercussions, from the age of 3 years old. She did not have secondary immunodeficiency (HIV, diabetes, immune suppressor treatment) or any familial history of primary immunodeficiency or similar lesions. She received all the childhood vaccines (BCG, MMR, diphteria, tetanus, poliovirus, and HBV) without any complications. She had no cardiac clinical symptoms and no electrocardiogram or echocardiographic abnormalities. No autoimmune symptoms had been reported. 

In terms of treatment, she has been treated with intravenous immunoglobulins substitution and antibiotic prophylaxis against opportunistic infections (Trimethoprim/Sulfamethoxazole). In addition, P3 had a treatment with imiquimod application on the skin and ample skin protection from UV radiation, allowing the clinical conditions to improve.

#### 2.1.3. Genotype

**Figure 3:**
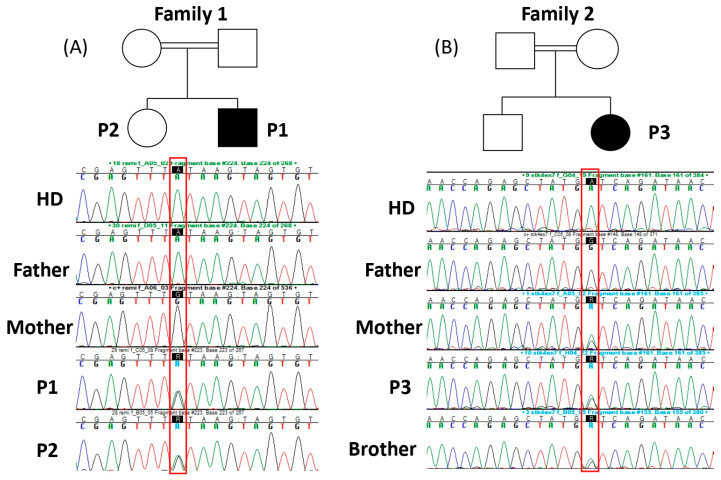
Pedigrees and familial segregation determined by Sanger sequencing of *STK4* variants in family 1 (**panel A**) and family 2 (**panel B**).

The consanguinity of parents and the clinical history of the three patients suggested an inborn error of immunity. To test this hypothesis, we performed genetic investigations in P1 and P3 by combining a deep sequencing array (using a custom-designed Illumina SNP array) with the 407 genes involved in inborn errors of immunity [25] and whole-exome sequencing. In both P1 and P3, we identified two predicted LOF homozygous variants in STK4, an essential splice site (c.1305+1G>A) in P1, and a premature stop codon (c.750G>A, p.W250*) in P3. We did not find any other candidate variants in the other genes related to syndromic EV. The familial segregation confirmed that P1, P2, and P3 were homozygous for their respective alleles, and their parents and healthy sibling were heterozygous (Figure 3A,B), suggesting that *STK4* was the disease-causing gene in these two families.

### 2.2. Immunological Phenotype

We then performed immunological analyses in both the clinical and research laboratories. In the clinical laboratory, P1 and P3 had a profound lymphopenia, with decreased T, B, and NK cell counts as compared with the normal range. In addition, P3 also had neutropenia. In contrast, P2, P1’s sister, had a normal count of T, B, and NK cells, with the exception of a mild CD4^+^ T cells lymphopenia. The HLA-DR expression was found to be normal on CD19^+^ and CD14^+^ cells in both siblings. In contrast, the immunoglobulin levels were normal in P1 and P2, with a slight hypogammaglobulinemia observed for P2. As for P3, there was a slight hypergammaglobulinemia and Hyper Ig E (Table 1 and Table 2).

**Figure 4:**
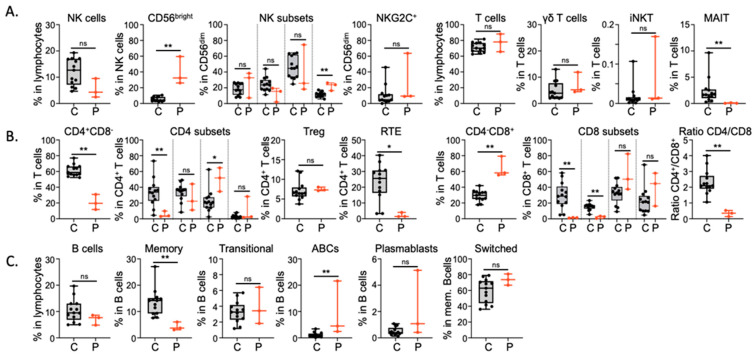
CyTOF analysis on peripheral blood. (**A**). Proportion of total and subsets of NK cells, of total T cells, total T cells, γδ T cells, iNKT and MAIT cells. (**B**). Proportion of total and naïve, central memory, effector memory and EMRA CD4^+^ T cells, T-regulatory, recent thymic emigrant (RTE) T cells and of total and naïve, central memory, effector memory and EMRA CD8^+^ T cell subsets. (**C**). Proportion of total and memory, transitional, Age-associated B cells (ABC), plasmablasts, and switched B cell subsets. Age-matched healthy donors (black), patients (red). *, *p* < 0.05; **, *p* < 0.01; ns, not significant *p* ≥ 0.05.

A deeper immunophenotyping has been carried out using Cytometry by Time of Flight (CyTOF) (Figure 4). The proportion of NK, CD3^+^, Treg and gamma-delta T cells in P1, P2 and P3 are in the normal range of healthy donors (Figure 4A). However, we observed a strong reduction in the CD4^+^ T cells proportion in T lymphocytes, an absence of MAIT cells, and a higher proportion of CD8^+^ T cells, leading to an inverted CD4/CD8 ratio. In addition, the proportion of recent thymic emigrant cells, as well as naïve CD4 and CD8 T cells, are strongly decreased. In contrast, memory CD4 and CD8 subsets are in the normal range or even increased in terms of proportion (Figure 4B). The B cell compartment also shows some major differences, such as a decrease in the memory B cells and an increase in the ABC subset (Figure 4C). Altogether, these immunological results are similar to the ones previously reported in patients with an STK4 deficiency.

## 3. Discussion

Human autosomal recessive (AR) STK4 deficiency was first reported in 2012 in seven patients with progressive T cell deficiency, and a broad range of infectious susceptibilities [26,27]. Since then, 29 additional cases of STK4 deficiency have been reported. All variants identified are loss-of-function [12,13,14,27,28,29,30,31,32,33,34,35,36,37,38,39,40]. The clinical phenotype related to STK4 deficiency is characterized by recurrent pulmonary bacterial infections, recurrent skin infections, including HPV warts (9 out 36) [12,14,27,28,40], including three with syndromic EV [12,13,14] and mucocutaneous candidiasis, but also chronic EBV infections. Auto-immune manifestations were reported in 13 cases [13,14,26,32,33,34,39,40]. The immunological phenotype of patients with STK4 deficiency is characterized by a profound CD4 lymphopenia due to a decreased proliferation, increased susceptibility to apoptosis, and the dysregulation of the transcription factor Forkhead box protein O1 (FOXO1) and its downstream targets in T cells. Leukocytes also show defective adhesion and chemotaxis [12,24,27,40,41].

We report here three children from two independent families with rare variants in STK4 deficiency and a broad clinical phenotype, including syndromic EV for two of them. Respiratory infections were noted in P2 and P3 as reported in many cases [42]. P1 had recurrent diarrhea with no pathogens identified. Gastroenteritis was already reported in patients with STK4 deficiency [40]. A negative deviation from the norm of growth curve was noted in the three patients reported here, as previously reported in [36].

In terms of immunologic phenotype, cytopenia is a common feature in STK4 deficiency, and we indeed observed it in the three patients reported here [40]. Furthermore, neutropenia is secondary to infections, autoimmunity, immunomodulatory agents, and chemotherapy [32,42]. In P3, neutropenia was developed during the infectious episodes. Allergic manifestations are also reported in STK4 deficiency (asthma, atopic dermatitis) [40]. Despite P3 having high hyper IgE, none of the patients reported here developed any allergic symptoms for now.

Autoimmune diseases were also associated with STK4 deficiency. The presence of autoantibodies, such as antinuclear and anticardiolipin antibodies, has been described in STK4 deficiency [13,14,26,32,33,35,39,40]. The autoimmunity in STK4 deficiency may also be due to the defective regulation of development and function of regulatory T cells through modulation of FOXO1/FOXO3. In our patients, no autoimmune manifestation has been reported so far. 

Some patients with STK4 deficiency are particularly prone to EBV-driven infections and EBV-induced lymphoproliferation [40], but this was absent in our patients. 

To conclude, we describe here three new clinical phenotypes related to STK4 deficiency. We treated our patients with IVIG, antibacterial prophylactic agents associated with imiquimod application on the skin, and skin protection from UV radiation for the EV patients. Although HSCT is the curative treatment in most CIDs, the survival rate after HSCT is about 50% in STK4 deficiency [38], and further studies are needed to recommend HSCT as a safe therapy for patients with STK4 deficiency.

## Figures and Tables

**Table 1:** Lymphocyte subpopulation counts.

Lymphocyte Subpopulations (N/mm3)	P1 (12 Years)	P2 (8 Years)	P3 (6 Years)	Normal Range (Age Matched)
**T cells**				
CD3^+^	771	2990	1489	1200–2600
CD4^+^	253	470	243	650–1500
CD8^+^	450	2330	1057	404–826
**B cells**				
CD19^+^	128	430	131	270–860
**NK cells**				
CD16^+^/CD56^+^	74	110	123	100–480

**Table 2:** Immunoglobulin levels.

Immunoglobulin Levels (g/L)	P1 (12 Years)	P2 (8 Years)	P3 (6 Years)	Normal Range (Age Matched)
IgG	11.62	5.17	17.3	6.10–16.16
IgM	0.49	0.46	2.09	0.22–2.40
IgA	1.99	3.21	2.17	0.84–4.99
IgE (UI/mL)	10	5.18	175.68	<100

## Data Availability

Upon request.
